# A Laboratory and Field Assessment of the Performance of Rebar Coatings

**DOI:** 10.3390/ma16124270

**Published:** 2023-06-08

**Authors:** Salah U. Al-Dulaijan

**Affiliations:** 1 Department of Civil and Environmental Engineering, King Fahd University of Petroleum & Minerals, Dhahran 31261, Saudi Arabia; sud@kfupm.edu.sa; 2 Interdisciplinary Research Center for Construction and Building Materials, King Fahd University of Petroleum & Minerals, Dhahran 31261, Saudi Arabia

**Keywords:** reinforcement corrosion, anti-corrosion coatings, steel primer, zinc-rich epoxy coating, cement-based epoxy coating, salt spray, accelerated corrosion evaluation

## Abstract

Deteriorating concrete structures are repaired to restore their load-carrying capacity and enhance their appearance. As part of the repair procedure, the corroded reinforcing steel bars are cleaned by sandblasting, and a protective coating is applied to protect them from further corrosion. Generally, a zin-rich epoxy coating is used for this purpose. However, there have been concerns about the performance of this type of coating in protecting the steel due to the formation of galvanic corrosion, thus necessitating the need for developing a durable steel coating. In this study, the performance of two types of steel coatings, namely a zinc-rich epoxy and cement-based epoxy resin coating, was investigated. The performance of the selected coatings was evaluated by conducting both laboratory and field experiments. In the field studies, the concrete specimens were exposed to a marine exposure site for more than five years. The salt spray and accelerated reinforcement corrosion studies indicated that the performance of the cement-based epoxy coating was better than the zinc-rich epoxy coating. However, there was no visible difference between the performance of the investigated coatings in the reinforced concrete slab specimens placed in the field. It is suggested to use cement-based epoxy coatings as steel primers based on the field and laboratory data developed in this study.

## 1. Introduction

The corrosion of reinforcing steel is a major problem in many concrete structures around the globe [1]. This problem becomes more significant in structures exposed to aggressive chloride environments [2]. Generally, the corrosion of steel will decrease the service life of RC structures and result in significant economic losses. The consequent impact of reinforcement corrosion includes the loss of bond between reinforcement and concrete, cracking of the concrete cover, and a decrease in the cross-sectional area of the steel [3,4]. The cost of corrosion damage is rapidly increasing (it has been estimated to be between 3 and 4% of GDP) [5,6]. A considerable proportion of this cost could be saved by using appropriate corrosion protection measures [7]. Further, the deteriorated concrete needs to be repaired to enhance the structural stability and aesthetics of a structure. The general procedure for repair is to remove the delaminated/spalled concrete, clean the steel bar, apply a bond coat to the concrete substrate and a steel primer to the steel, repair the vacant area by applying new concrete or mortar, and coat the repair surface with a protective coating. Different reinforcing bars can be used to mitigate the corrosion of steel in concrete. These include fiber-reinforcing polymer (FRP) bars, stainless-steel clad steel bars, and coated rebars [8]. In particular, the coating and cladding techniques have demonstrated an improved corrosion resistance of steel reinforcement, although the use of stainless-steel clad steel bars is preferred for reinforced concrete structures, especially in extremely corrosive marine environments or when long service lives are required [9,10,11].

Anti-corrosion coatings are applied on the old steel after cleaning it by sandblasting [12,13,14,15,16]. Generally, organic coatings are classified into polymeric materials or polymer-modified materials (e.g., mixed with cement or fly ash) [17,18,19,20]. These coatings mainly work through two mechanisms: forming a physical barrier or chemically binding the chloride ions [21,22]. Thus, the underlying steel can be protected from corrosion by applying coatings that can form a passivating environment around the rebars or provide physical protection. Zinc-rich epoxy coating is widely used for corrosion protection since it is believed that the zinc in the coating would become sacrificial in the event of future corrosion of the steel bars in the repaired portion [23,24,25]. However, there are chances of crevice corrosion of the steel at junctions between the repaired and unrepaired areas due to the difference in the environment around the steel bars at these two locations. Additionally, there are chances of hydrogen evolution due to the dissolution of zinc at high pH of the concrete pore solution. As a result, a few new cement-based rebar primers have been developed recently [26,27,28]. It is claimed that these primers will eliminate the problem of crevice corrosion at the junction of repaired and sound areas and the galvanic corrosion of reinforcing steel.

Pei et al. [7] investigated the corrosion protection of cementitious coatings on steel rebars exposed to sodium chloride. The results in terms of visual inspection, corrosion potentials, and time to corrosion initiation indicated that the application of coatings improved corrosion resistance (the onset of corrosion was delayed by around 2–4 months during which the corrosion potential was in a non-active range). The experimental results based on the linear polarization resistance technique and electrochemical impedance spectroscopy showed that the cement-based coating was superior to the epoxy coating in a 5% NaCl solution [29]. It was reported that the high alkalinity of the cement and its good physical shielding properties were the main reasons behind the improved anti-corrosion performance of cement-based coatings [29]. Wang et al. [30] investigated the anti-corrosion performance of a magnesium–potassium–phosphate cement-based coating on steel. The results revealed that the developed coating has good anti-corrosion performance. In addition, it was reported that the anti-corrosion ability of the coating was effectively enhanced when it was modified with silica fume. In another study, Zhang et al. [31] investigated the anti-corrosion performance of magnesium potassium phosphate cement applied on steel bars. The results indicated that the proposed coating types performed well in protecting the steel bars, and their effectiveness exceeded the coating prepared with ordinary Portland cement. Recently, graphene-based anti-corrosive coatings have been investigated [32]. Generally, nanocomposite-based coatings are promising due to their significant protectiveness against the corrosion of steel in concrete and their long-term anti-corrosion behavior.

Chen et al. [26] studied the corrosion resistance of cementitious coatings modified with Ca–Al–NO_3_-layered double hydroxides (Ca-LDH). It was reported that the chloride threshold of the modified cementitious coating increased by 0.06–0.07 M, depending on the Ca-LDH content. The improvement in the chloride tolerance level was attributed to the protective effect of the cement paste coating and the ability of Ca-LDH to bind with chloride ions. Arya et al. [14] reported that the application of a cement–polymer composite coating on the rusted steel rebars (as-received rebars) reduced the service life to around 40%. In addition, Jorge et al. [33] reported that the loss in bond strength due to the application of cementitious coatings on uncleaned steel rebars can be as high as 40%. Kamde and Pillai [17] concluded that sandblasting the steel coated with a cement–polymer composite coating increased the chloride threshold (by about 50%), and hence the desired service life was achieved. Therefore, it is recommended to sandblast the steel bars prior to the application of the steel primer to increase the bond and prevent the premature initiation of corrosion. Harilal et al. [34] proposed a ternary coating consisting of fly ash, nanoparticles, and inhibitors. Based on the test results of coated bars exposed to an aggressive chloride environment, it was reported that the composite coating delayed the onset of corrosion. Since there is not much data to validate the effectiveness of cement-based coatings in retarding reinforcement corrosion, it is necessary to evaluate the performance of cement-based coating with reference to the zinc-rich coating.

The reported study was conducted to assess the performance of cement-based and zinc-rich epoxy primers in mitigating corrosion by conducting laboratory and field studies. In the laboratory program, two types of specimens, namely cylindrical concrete specimens with a central reinforcing bar and steel coupons, were prepared. Concrete slab specimens that were repaired using selected steel primers were exposed in the field.

## 2. Research Methodology

To study the effectiveness of the selected primers in concrete repair, both laboratory and field experiments were conducted. Under the laboratory program, two types of specimens, cylindrical concrete specimens with a central reinforcing steel bar and steel coupons, were used. The field specimens were prepared with coated and uncoated steel bars. The repaired concrete slab specimens prepared with coated bars were exposed to field conditions.

### 2.1. Materials and Concrete Mix

The concrete mixture was prepared with Type I cement conforming to ASTM C150 [35]. Dune sand with a specific gravity and water absorption of 2.64 and 0.57%, respectively, was used as a fine aggregate. Crushed limestone with a maximum size, specific gravity, and water absorption of 12.5 mm, 2.43, and 2.57%, respectively, was used as a coarse aggregate. The concrete mixtures were prepared with a cement content of 370 kg/m^3^ and a water-to-cement ratio and a coarse-to-fine aggregate ratio of 0.4 and 1.6, respectively. Deformed reinforcing steel conforming to ASTM A615 [36] was used in the preparation of reinforced concrete specimens for both laboratory and field evaluation. Mild steel plates were used for accelerated salt spray tests.

The steel bars and steel plates were coated with two types of coatings, namely a zinc-rich epoxy and three-component cement-based epoxy resin. Table 1 shows the details and properties of the used coatings according to the manufacturer’s specifications.

### 2.2. Laboratory Investigation

Two types of laboratory specimens, namely cylindrical concrete specimens with a central reinforcing bar and steel coupons coated with the selected steel primers, were prepared. Slab specimens were prepared for field exposure. The specimens were labeled, as shown in Table 2, to indicate the type of coating applied to the steel bars.

#### 2.2.1. Casting and Curing of Cylindrical Concrete Specimens

**Figure 1:**
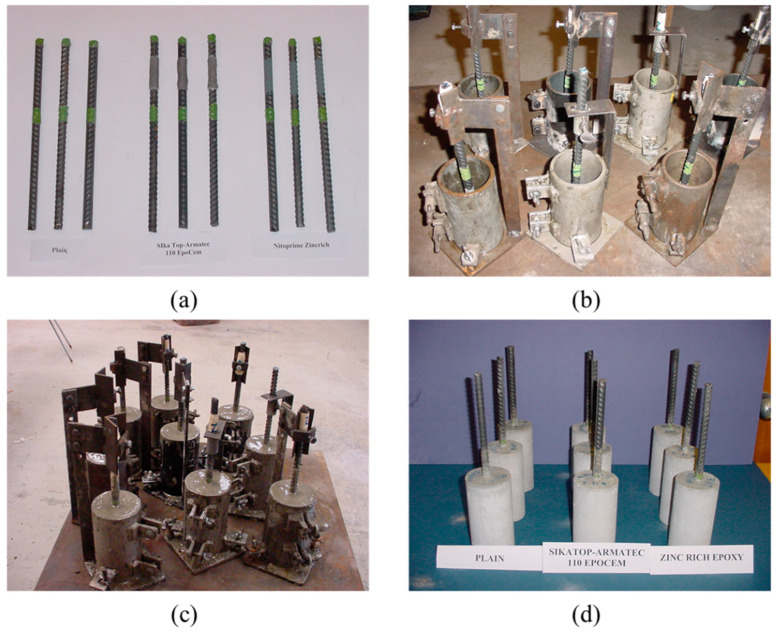
The sequence of preparing the concrete specimens: (**a**) uncoated and coated steel bars; (**b**) steel molds with centered uncoated and coated steel bars; (**c**) steel molds filled with concrete; and (**d**) concrete specimens with uncoated and coated steel bars after demolding.

Cylindrical concrete specimens, 75 mm in diameter and 150 mm high, with a central reinforcing steel bar, were prepared. The steel bars were cleaned by sandblasting and coated with the selected steel primers. The control specimens with uncoated steel bars were also prepared. Figure 1 shows the sequence of preparing the concrete specimens.

#### 2.2.2. Testing of Cylindrical Concrete Specimens

**Figure 2:**
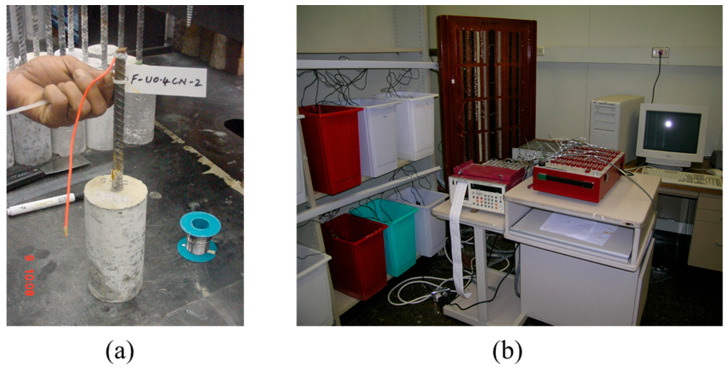
(**a**) Electrical lead wires being soldered to a steel bar and (**b**) accelerated corrosion test setup.

After curing, the cylindrical concrete specimens were prepared for accelerated corrosion testing by firstly soldering wire leads to the reinforcing steel bars. Figure 2a shows wire leads being soldered to the steel bar.

The cylindrical concrete specimens were partially immersed in a 5% NaCl solution (representing the chloride concentration in the seawater), and the reinforcing steel was corroded by impressing an anodic potential of 4 volts. Figure 2b shows the setup used for accelerating reinforcement corrosion. The current required to maintain the anodic voltage of 4 V was recorded at periodic intervals using a data acquisition system. Triplicate specimens were used for each condition of the steel bars. The time-current curves were utilized to assess the time to initiation of cracking in concrete due to reinforcement corrosion. The specimen was assumed to have cracked when a significant change in the slope of the time-current curve was noticed [37].

#### 2.2.3. Preparation and Testing of the Steel Panels

A salt spray exposure study was conducted on the steel panels coated with the selected coatings. The coated steel panels were exposed to salt spray for 1000 h in a fog chamber, according to ASTM B117 [38]. The evaluation of the coated panels exposed to the salt spray for 1000 h was carried out according to ASTM D714 [39] and ASTM D1654 [40].

**Figure 3:**
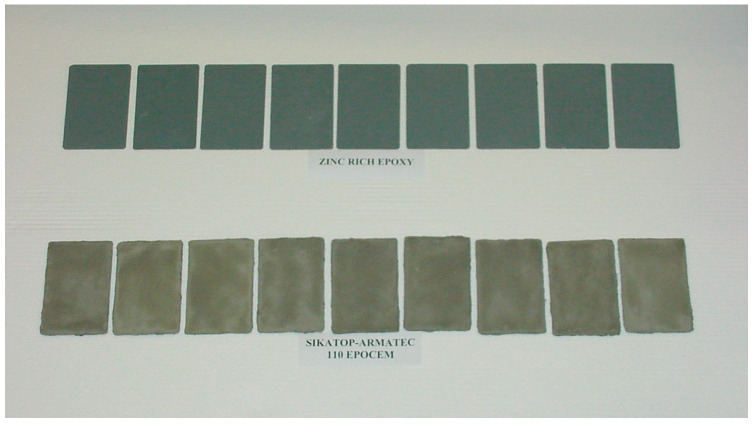
Coated steel panels.

The steel test panels were 150 × 100 × 3 mm in size. Two sets of panels were prepared. One set of panels was coated with the cement-based coating, while the other set was coated with the zinc-rich epoxy coating. Three coated panels from each set were scribed, while the other three panels were not scribed. Figure 3 shows the coated steel panels. The panels were mounted in the fog chamber on a perforated Perspex (Plexiglas) tray. The panels were placed on the tray at an angle of inclination of 15 degrees to the vertical. A suitable distance was kept between the panels to permit the free settling of the fog. The salt spray test was conducted according to ASTM B117 [38], and the test conditions are shown in Table 3.

After the salt spray exposure, the coated steel panels were carefully retrieved from the chamber and gently rinsed in warm distilled water (38–40 °C) to remove salt deposits and immediately dried by a stream of clean compressed air. This was followed by an instant examination according to ASTM D1654 [40], Procedure A, and ASTM D714 [39].

### 2.3. Field Investigations

#### 2.3.1. Preparation of Field Specimens

**Figure 4:**
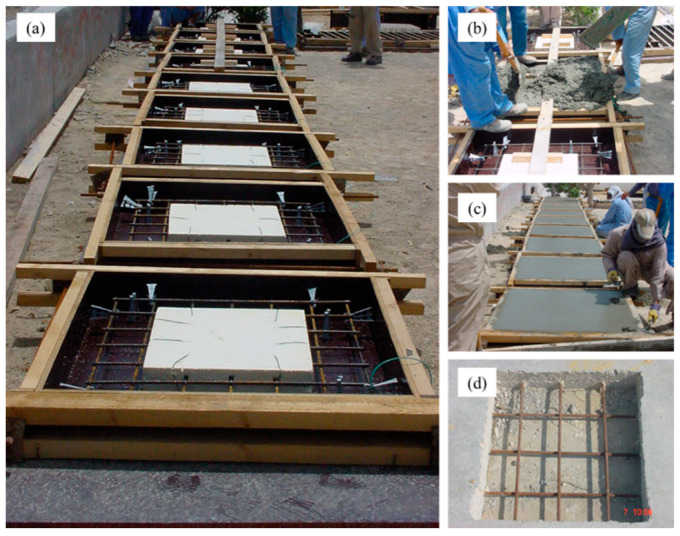
Preparation of field specimens: (**a**) reinforcing steel bars in the molds with the Styrofoam boards to create cavities; (**b**) pouring and consolidation of the concrete in the molds; (**c**) finishing the concrete in the molds; and (**d**) corroded steel bars in the concrete specimens.

Reinforced concrete slab specimens, measuring 1.0 × 1.0 × 0.15 m, contaminated with 1.5% sodium chloride to resemble contaminated concrete, were prepared for field exposure. A total of nine reinforced concrete slab specimens were prepared for field exposure. Cavities were created in the slab specimens for later repair. These cavities were created by placing a Styrofoam board in each mold prior to the casting of concrete, as displayed in Figure 4a. Figure 4b,c show the concrete specimens being cast on the exposure site. After casting, the specimens were cured by covering them with wet burlap for 14 days.

**Figure 5:**
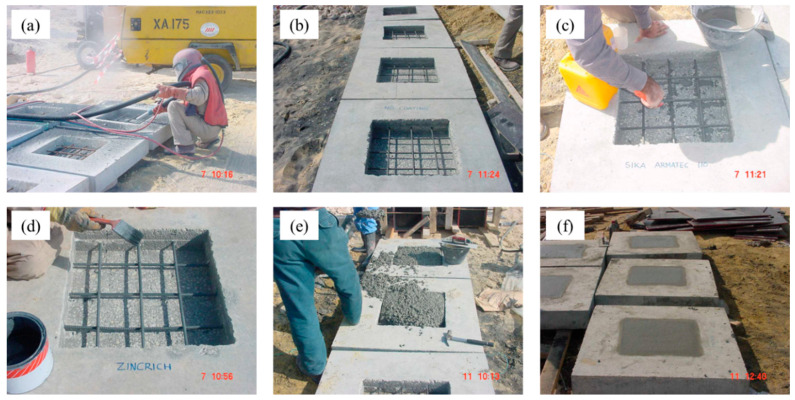
Coating application: (**a**) cleaning of the corroded steel bars by sandblasting; (**b**) concrete specimens after the cleaning of the corroded steel bars; (**c**) cement-based coating being applied on the steel bars in one of the specimens; (**d**) zinc-rich epoxy coating being applied on the steel bars; (**e**) cavities being filled with concrete; and (**f**) concrete slab specimens after repairing.

After the curing of the concrete specimens, the steel bars in the induced cavities were deliberately corroded by spraying salt water until significant corrosion was noted. Figure 4d shows a concrete specimen with corroded reinforcement. The corroded reinforcing steel bars were then cleaned by sandblasting, as shown in Figure 5a. The concrete specimens with clean reinforcements are shown in Figure 5b. After cleaning, the exposed steel bars were coated with the selected coatings. Figure 5c,d show the coating of the steel bars. After the coating had dried, the cavities were filled with concrete. Figure 5e shows the process of filling the cavities with concrete. Figure 5f shows the slabs after the finishing process, in which the slabs were covered by wet burlap for the curing of the repaired area. Control specimens without any coating on the steel bars were also prepared for the sake of relative comparison.

#### 2.3.2. Exposure, Monitoring, and Testing of the Field Specimens

After curing, the slab specimens were placed in the splash zone of a marine environment in the Arabian Gulf. The average and maximum annual relative humidity and air temperature at the exposure site were 50%, 95%, 26.7 °C, and 47 °C, respectively. The average chloride concentration of the seawater was 26,000 ppm.

**Figure 6:**
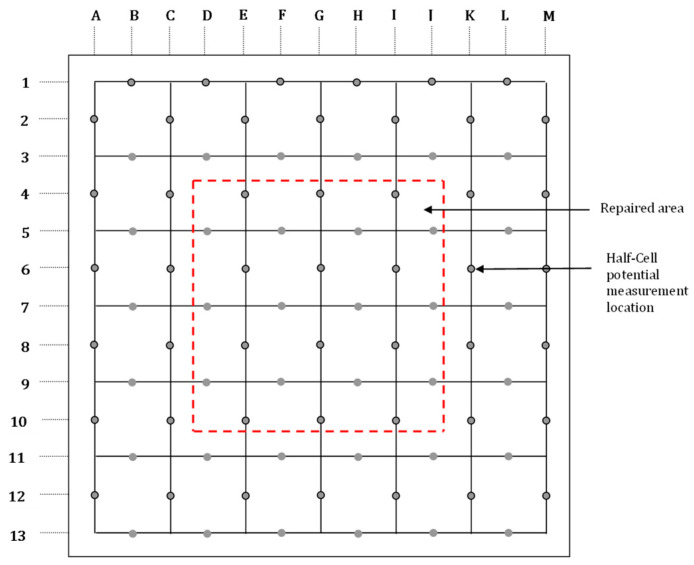
Grid showing the points for measuring the corrosion potentials on the concrete slab specimens.

The specimens were exposed to the natural low and high tides of the sea. Reinforcement corrosion in the slab specimens was monitored every four months by measuring corrosion potentials at pre-marked locations on both the repaired and unrepaired areas for 2267 days (approx. 6.2 years). For this purpose, a grid was marked on the concrete specimens, as shown in Figure 6. Reinforcement corrosion was monitored by measuring the corrosion potentials at the marked locations. The potentials were measured by connecting the steel bars to the negative terminal of a digital multimeter, while the positive terminal was connected to a copper–copper sulfate reference electrode.

## 3. Results and Discussion

### 3.1. Analysis of Laboratory Investigations

This section is divided into two parts. The first is related to the analysis of the results of the tests conducted on the steel panels and the second presents the analysis of the results of the accelerated corrosion of the cylindrical concrete specimens.

#### 3.1.1. Salt Spray Results on Coated Panels

The exposed specimens were rated according to ASTM D1654 [40] Procedure-A to determine the extent of failure. The corrosion creepage rating at the scribes was selected for both sets of coated panels. The mean failure distance from the scribe was 0 mm, and the rating number was 10.

**Figure 7:**
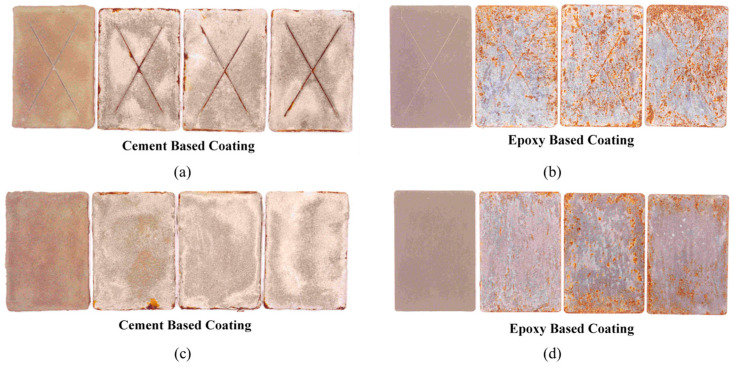
(**a**) Scribed steel panels coated with cement-based coating and (**b**) scribed steel panels coated with zinc-rich epoxy coating. (**c**) Unscribed steel panels coated with cement-based coating and (**d**) unscribed steel panels coated with zinc-rich epoxy coating. All the coated steel panels were exposed to salt spray for 1000 h.

The visual inspection of the exposed surfaces of the specimens was conducted to evaluate the extent of deterioration of the coating after exposure to the salt spray for 1000 h. There was no blistering and/or delamination of the coating or along the scribed lines on any of the coated steel panels. However, many corrosion spots, distributed all over the surface, were noted on the steel panels coated with the zinc-rich epoxy coating. A few spots of corrosion were also noted on the steel panels coated with the cement-based coating. However, the coating was intact on all the coated panels. Figure 7 shows the scribed and unscribed steel panels coated with the cement-based and zinc-rich epoxy coating before and after exposure to the salt spray. Table 4 and Table 5 summarize the results of the visual examination of the steel panels coated with cement-based coating and zinc-rich coating, respectively.

The anti-corrosion performance of different coating types on the steel panels was investigated by several researchers. Al-Tholaia et al. [41] investigated three types of coatings, namely red oxide, zinc primer, and epoxy resin, for corrosion protection on steel panels. It was reported that the epoxy coating performed well against chloride-induced corrosion. Kalendová et al. [42] investigated the anti-corrosion properties of polyaniline coatings produced by epoxy binders on steel panels. The results demonstrated that the polyaniline coating enhanced the anti-corrosion properties of the tested panels after exposure to a salt mist cabinet. Wang et al. [30] studied the effect of adding silica fume to the magnesium potassium phosphate cement-based coating. The modified coating showed a good anti-corrosion effect on steel panels tested in a 3.5% sodium chloride solution. Compared with the previous research, our results desaturate the good anti-corrosion performance of the tested coatings (cement-based and zinc-rich epoxy coatings). However, further research is recommended to be conducted on the adhesion performance between the steel substrate and the coating.

**Table 4:** Results of a visual examination of steel panels coated with the cement-based coating.

Panel	ASTM Standard	Rating
Coated and scribed	D1654 [40] Procedure A	10
Coated and scribed	D714 [39]	No blistering or delamination of the coating and no corrosion spots
Coated and scribed	D610 [43]	Rust grade: 2 (0% of surface rusted)
Coated and unscribed	D1654 [40] Procedure B	10
Coated and unscribed	D714 [39]	No blistering or delamination of coating and no corrosion spots
Coated and unscribed	D610 [43]	Rust grade: 2 (0% of surface rusted)

**Table 5:** Results of a visual examination of the steel panels coated with the zinc-rich epoxy coating.

Panel	ASTM Standard	Rating
Coated and scribed	D1654 [40] Procedure A	10
Coated and scribed	D714 [39]	No blistering or delamination of coating but lots of corrosion spots
Coated and scribed	D610 [43]	Rust grade: 2 (33% of surface rusted)
Coated and unscribed	D1654 [40] Procedure B	10
Coated and unscribed	D714 [39]	No blistering or delamination of coating but lots of corrosion spots
Coated and unscribed	D610 [43]	Rust grade: 2 (33% of surface rusted)

#### 3.1.2. Results of Accelerated Corrosion Tests

**Figure 8:**
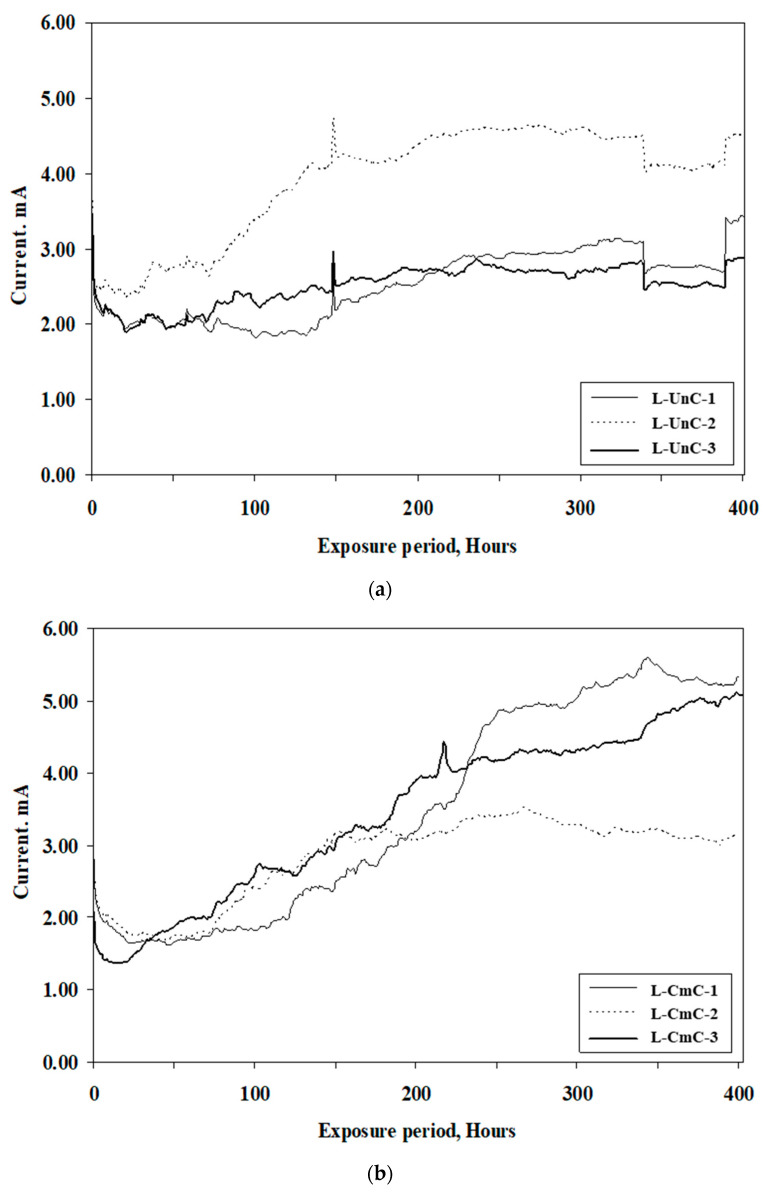

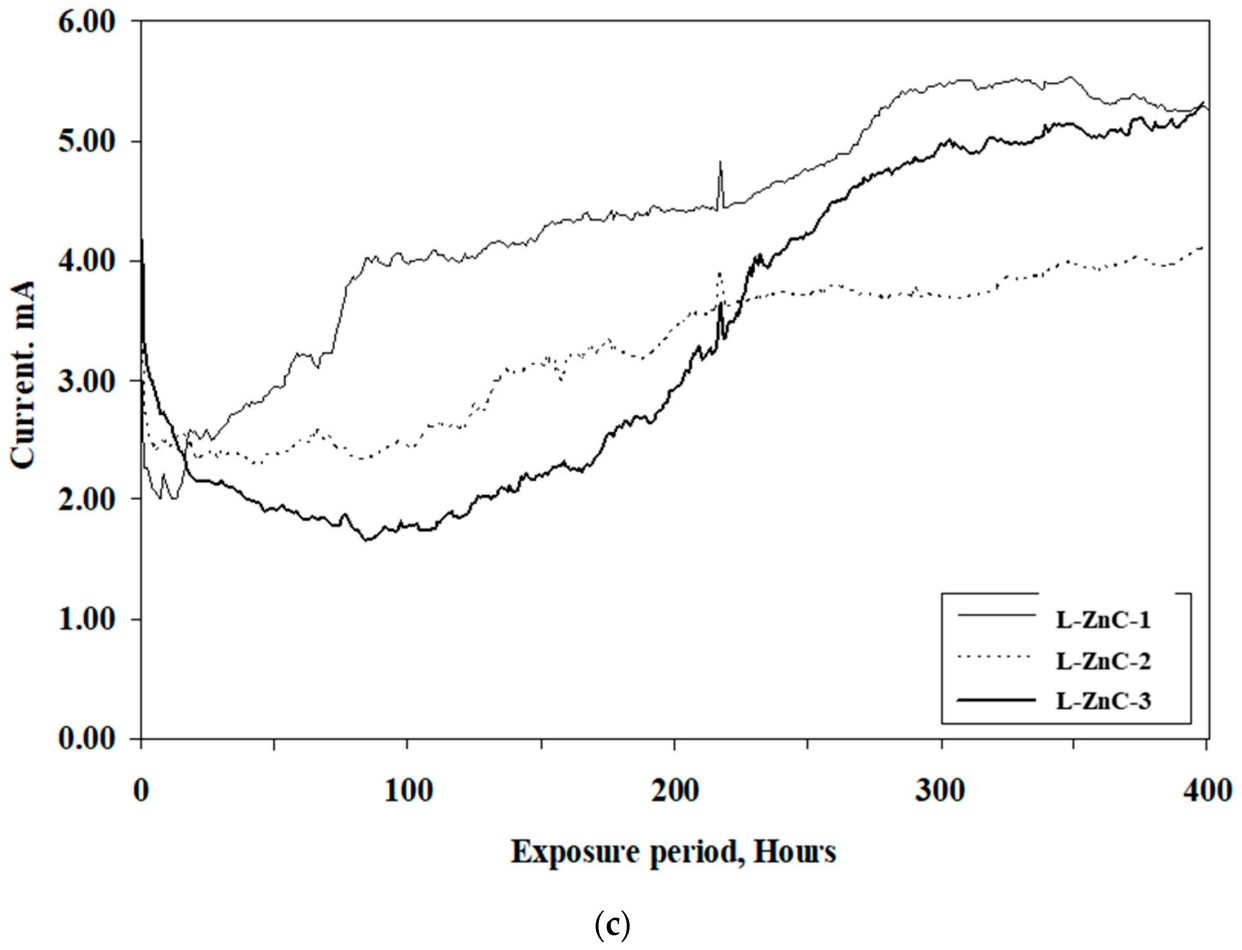
Time-current curves for cylindrical concrete specimens: (**a**) uncoated steel bars; (**b**) steel bars coated with cement-based coating; and (**c**) steel bars coated with zinc-rich epoxy coating.

The time-current curves for the uncoated steel bars and those coated with the selected coatings are shown in Figure 8a–c. These curves were utilized to determine the time to crack of the concrete specimens subjected to an impressed anodic potential of 4V. As stated earlier, the time to crack was taken as the point at which a significant change in the slope of the current-time curve was noticed. The time to crack of the concrete specimens, as calculated from the time-current curves, is summarized in Table 6. The average value of measurements conducted on three specimens is also reported. The average time to crack in the concrete specimens with uncoated steel bars was 113 h, and it was 282 and 110 h, respectively, in the bars coated with cement-based and zinc-rich epoxy coatings. Thus, the cement-based coating delayed the time to crack by about 2.5 times more than the uncoated specimens, while the zinc-rich epoxy coating showed a negligible effect, as its performance was almost similar to that of the uncoated rebars. This highlights the beneficial effect of the cement-based coating in extending the service life of RC structures. The superior performance of the bars coated with the cement-based coating may be attributed to the physical protection provided by the coating and, at the same time, maintaining the high pH, thereby minimizing galvanic and crevice corrosion [7,44].

### 3.2. Analysis of Field Results

**Figure 9:**
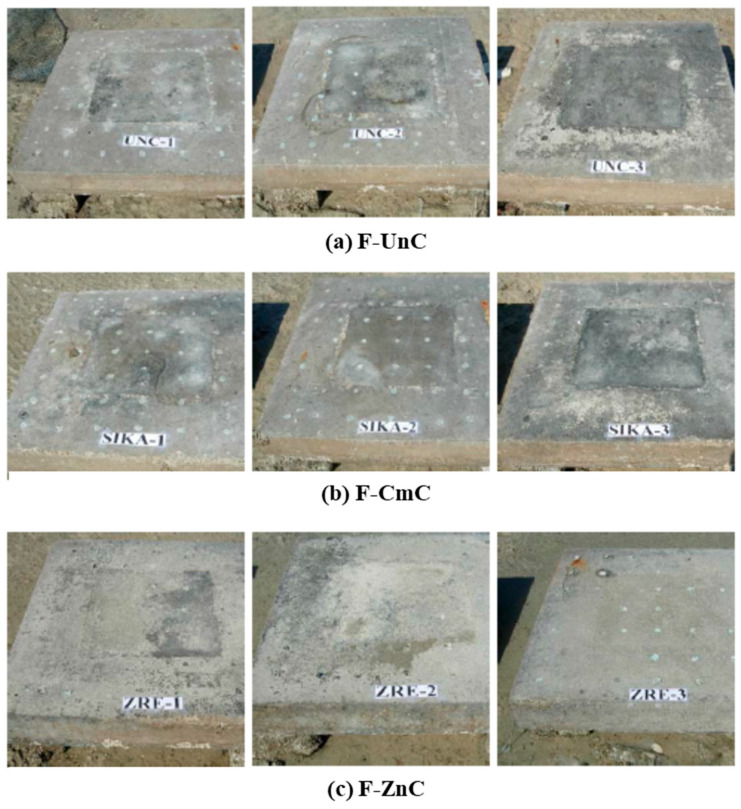
Concrete specimens placed in the tidal zone: (**a**) specimen F-UnC; (**b**) specimen F-CmC; and (**c**) specimen F-ZnC.

Figure 9 shows the slab specimens with uncoated and coated steel bars placed in the tidal zone of a marine environment. After five years of exposure, minor longitudinal cracks were noted in almost all the specimens. Rust stains were also noted in some of the specimens. These cracks were, however, not at the interface between the repaired and unrepaired areas. The cracks were noted mostly at the edges of the specimens. Since these specimens were in the tidal zone, they were intermittently exposed to seawater, and since sufficient oxygen is available at the edges, corrosion of reinforcing steel at the edges is accelerated compared to the inner parts of the specimens. Further, the depth of carbonation measured after about six years of field exposure was very low (less than 5 mm); thus, corrosion is mainly caused by the chloride ions.

**Figure 10:**
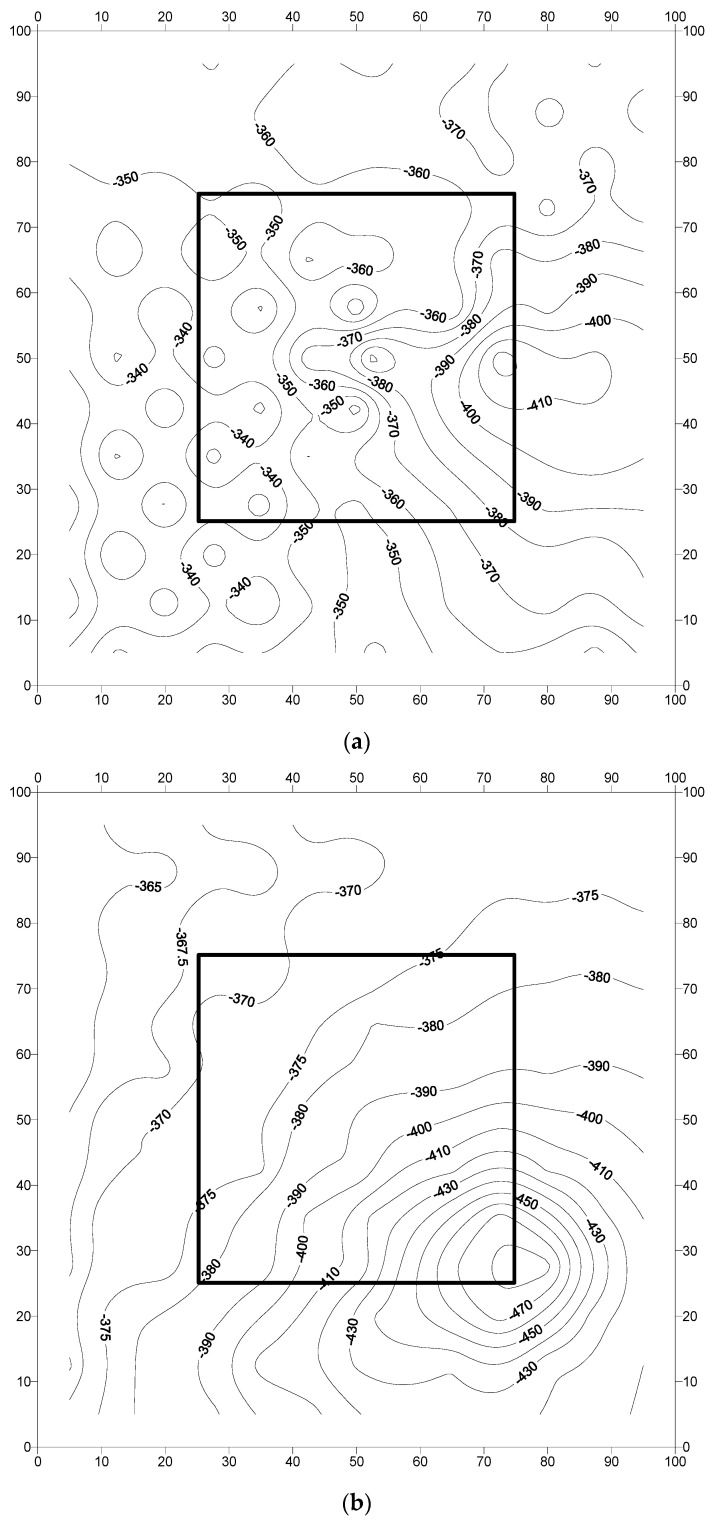

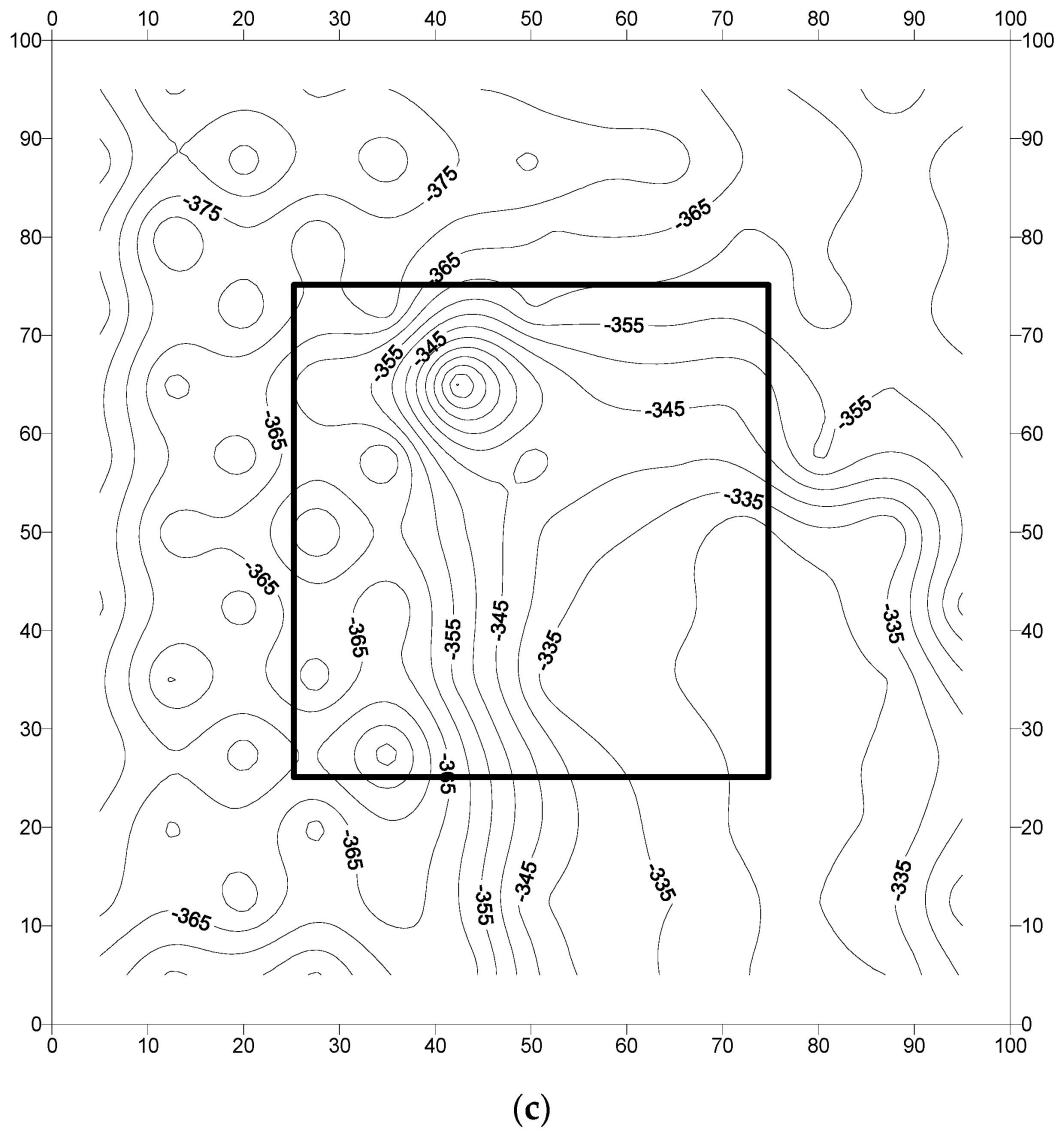
Potential contours in the reinforced concrete slab specimens prepared with uncoated steel bars: (**a**) specimen F-UnC-1; (**b**) specimen F-UnC-2; and (**c**) specimen F-UnC-3. Potential values are in mV CSE.

**Figure 11:**
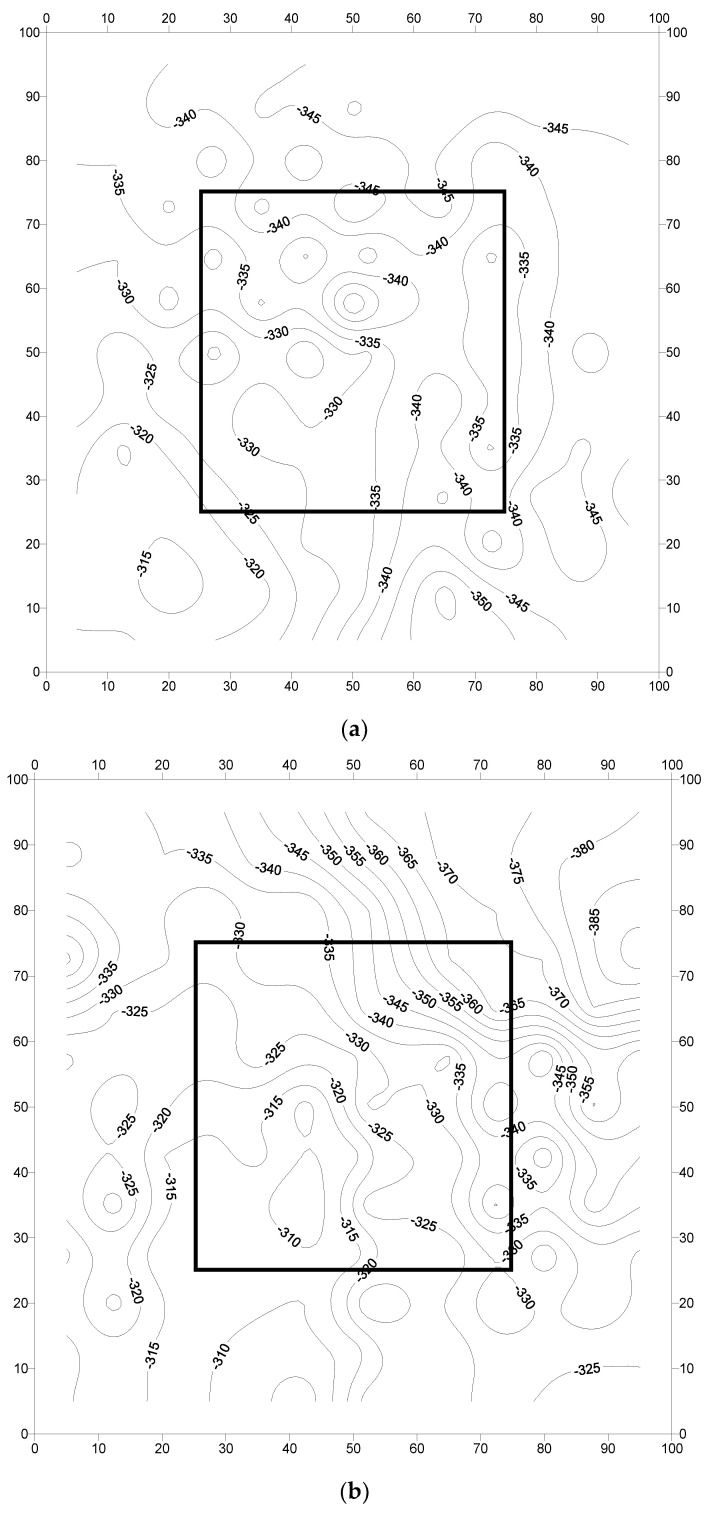

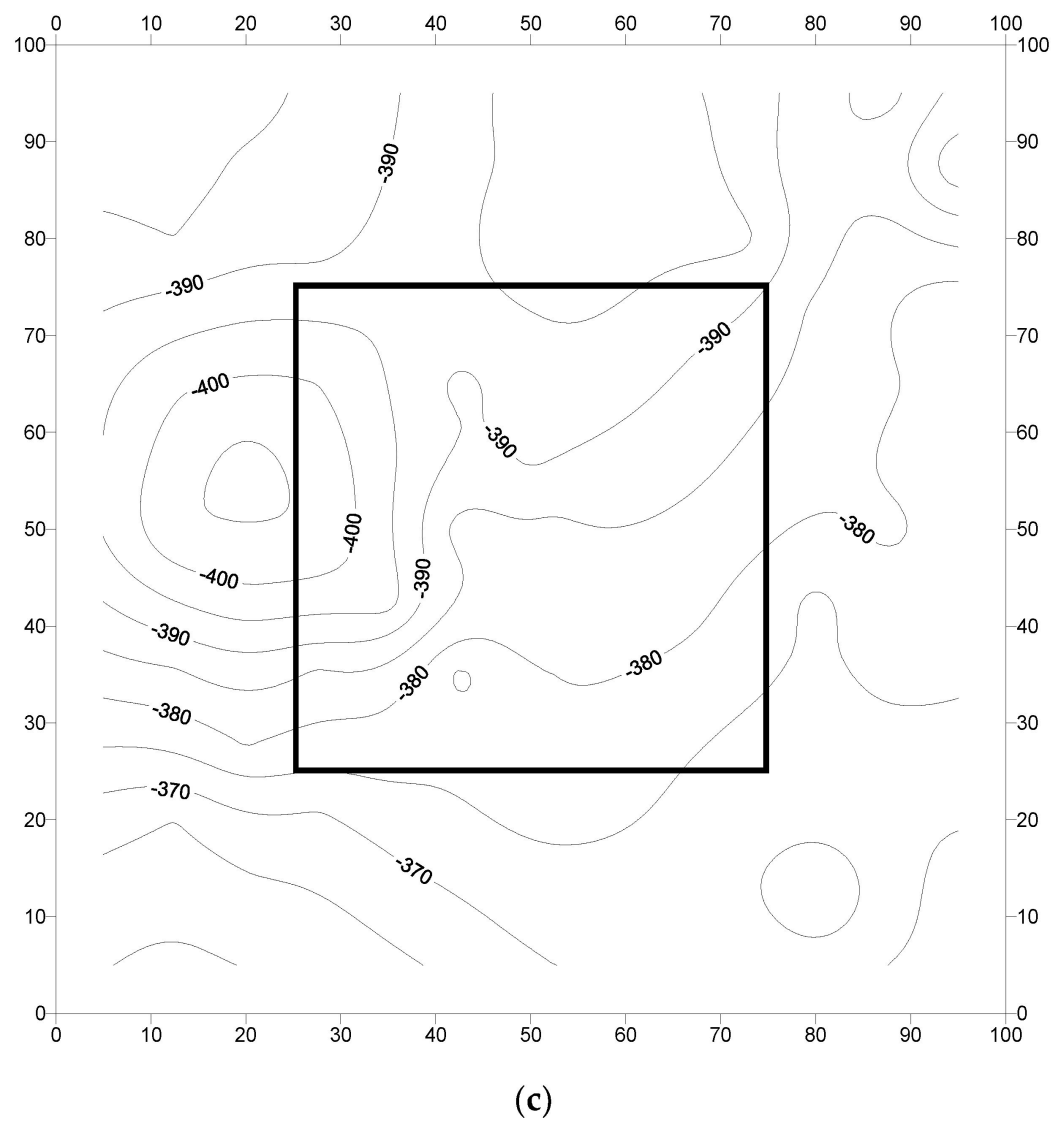
Potential contours in the reinforced concrete slab specimens prepared with steel bars coated with a cement-based coating: (**a**) specimen F-CmC-1; (**b**) specimen F-CmC-2; and (**c**) specimen F-CmC-3. Potential values are in mV CSE.

**Figure 12:**
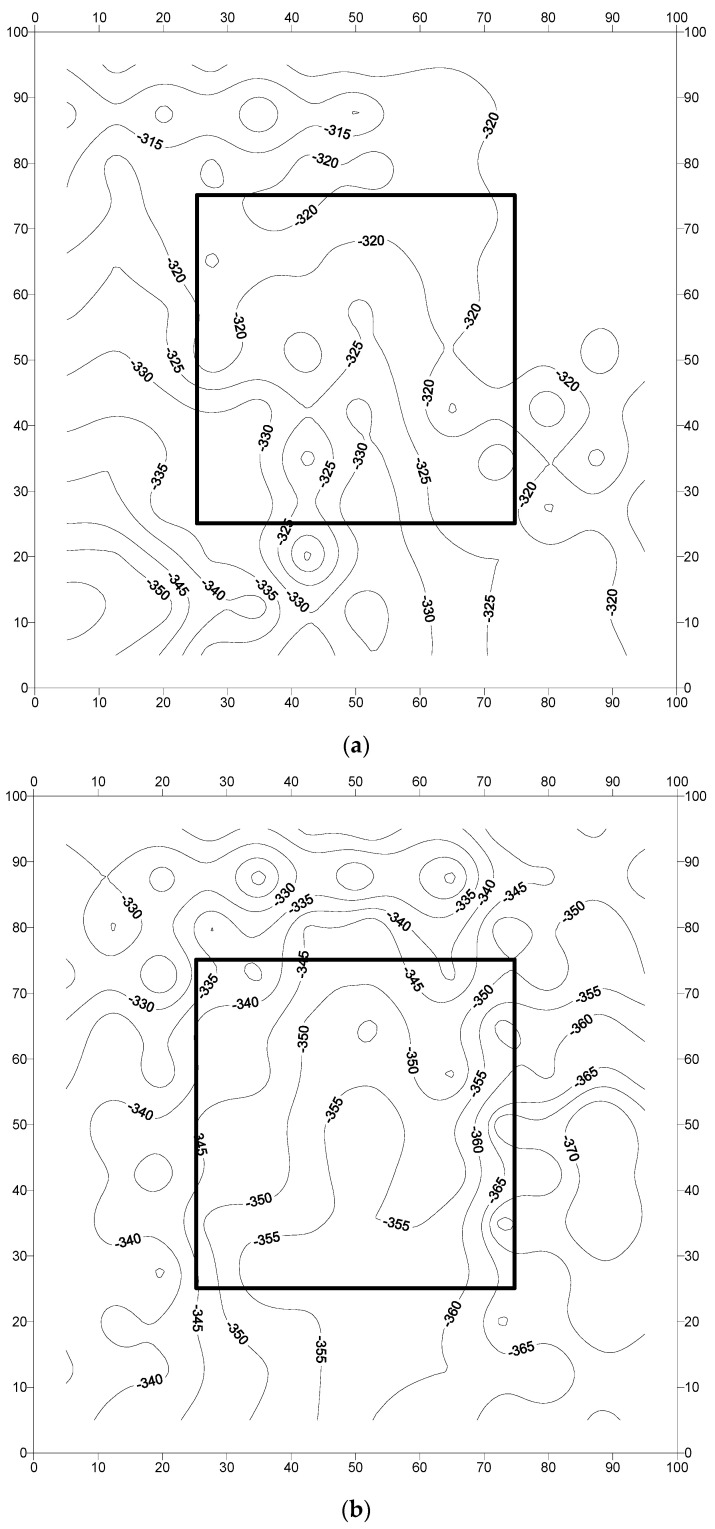

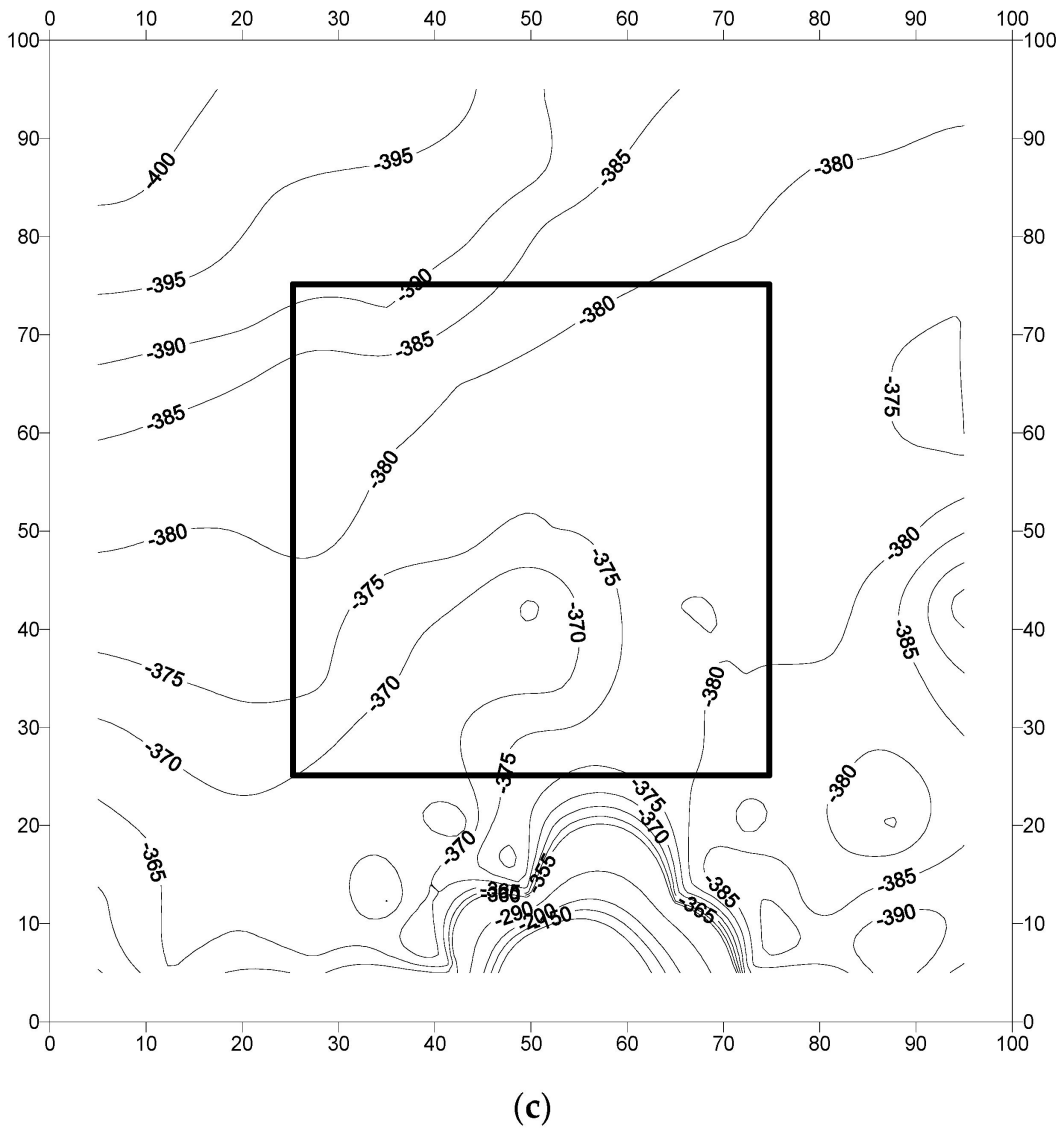
Potential contours in reinforced concrete slab specimens prepared with steel bars coated with the zinc-rich epoxy coating: (**a**) specimen F-ZnC-1; (**b**) specimen F-ZnC-2; and (**c**) specimen F-ZnC-3. Potential values are in mV CSE.

The corrosion potentials measured at pre-marked locations on the concrete slab specimens are presented in Figure 10, Figure 11 and Figure 12. Generally, the potentials were low (less negative) at the early stages and then started to increase with the period of exposure. The corrosion potentials after 240 days were generally more negative than the threshold value of −350 mV CSE, according to ASTM C876 [45]. The low values do not necessarily indicate the initiation of corrosion, as the presence of moisture may influence the resistance of the concrete [7]. The following paragraphs discuss the potential behavior of each specimen.

The potential contour plots for concrete specimens with uncoated steel bars are shown in Figure 10a–c. In specimen F-UnC-1 (Figure 10a), the potentials varied from −340 to −410 mV CSE. The potentials were generally uniformly distributed, with exceptional localization of the corrosion activity. The corrosion potentials in specimen F-UnC-2 (Figure 10b) were in the range of −360 to −480 mV CSE, and they were uniformly spread throughout the specimen. However, a steep potential gradient, indicating the chances of pitting corrosion, was noted at the interface between the old and the repaired area in the southeast corner of the specimen. In specimen F-UnC-3 (Figure 10c), the corrosion potentials were in the range of −295 to −375 mV CSE. A steep gradient in the potential readings was noted on the northwest side of the repair area, indicating the chances of pitting corrosion at that location.

The potential contours for the concrete specimens with reinforcing steel coated with the cement-based coating are shown in Figure 11a–c. The potential contours for specimen F-CmC-1, specimen F-CmC-2, and specimen F-CmC-3 were in the range of −315 to −345, −315 to −390, and −350 to −390 mV CSE, respectively. Generally, the corrosion was uniform, with isolated spots of localized corrosion in specimen F-CmC-1 (Figure 11a). Steep gradients in the potentials were not observed in this specimen. In specimen F-CmC-2 (Figure 11b), a uniform distribution of the potentials could be noted. The potentials in specimen F-CmC-3 (Figure 11c) were evenly distributed, except for some chances of pitting corrosion on the western side of the repair junction.

Figure 12a–c depicts the corrosion potential contours for reinforced concrete specimens prepared with steel bars coated with a zinc-rich epoxy. The corrosion potentials in specimens F-ZnC-1, F-ZnC-2, and F-ZnC-3 were in the range of −310 to −355, −320 to −370, and −150 to −400 mV CSE, respectively. As shown in Figure 12a, the potential variation was uniform in specimen F-ZnC-1, except at a few locations where a change in the potential gradient was noticed. The gradient was not steep enough to indicate the chances of pitting corrosion. The variation in the potential corrosion in specimen F-ZnC-2, shown in Figure 12b, was also uniform. However, a change in the potential in the order of 10 to 15 mV is noted at several locations. In specimen F-ZnC-3 (Figure 12c), the variation in the potentials was uniform in all the parts of the specimen, while a steep change in the potential gradient was noted in the lower part of the specimen. This steep gradient was not at the interface of the repaired and old concrete, indicating that it is not due to the formation of incipient anodes at the interface.

The data in Figure 10, Figure 11 and Figure 12 (corrosion potential contours after 2267 days of exposure to a splash zone in a marine environment) do not show any indication of the formation of incipient anodes at the interface of the repaired area and old concrete due to the application of the cement-based or zinc-rich epoxy coating. In addition, the corrosion potential data in the concrete slab specimens did not exhibit any explicit difference in performance between the uncoated and coated steel bars. Similarly, there was no significant difference in the performance of the selected coatings, namely the cement-based and zinc-rich coating. Such behavior may be attributed to the size of the specimens, which was large and representative of the real-life scenario. Therefore, long-term monitoring of the slab specimens is highly desirable to further ascertain the performance of the two selected coatings.

**Figure 13:**
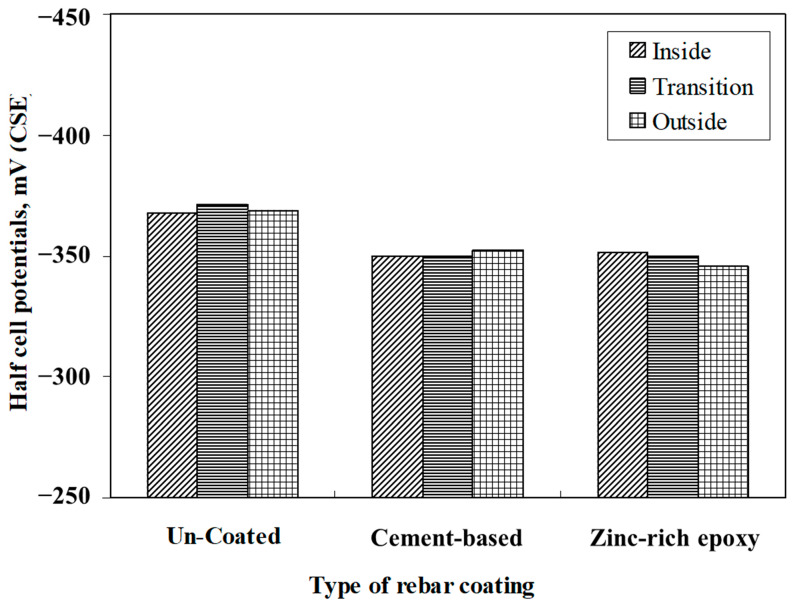
Average corrosion potentials in the concrete specimens with the uncoated bars and those coated with cement-based and zinc-rich epoxy coatings after 2267 days of exposure to a splash zone in a marine environment.

Figure 13 depicts the average corrosion potentials in the interior, exterior, and transition zones of the slab specimens after 2267 days of exposure to the splash zone in a marine environment. These data do not show a big difference in the average potential values in the concrete specimens with uncoated steel bars and those coated with either the cement-based coating or zinc-rich epoxy coating. Moreover, the average potentials in the interior, exterior, and transition zones were not significantly different. This indicates that the application of either a cement-based or zinc-rich epoxy coating did not induce corrosion at the junction of the repaired and old concretes.

## 4. Conclusions

An experimental investigation, comprising both laboratory and field studies, was conducted to assess the performance of cement-based and zinc-rich epoxy coatings. The following conclusions can be drawn based on the results of the laboratory and field studies:

i. There was no blistering and/or delamination of the coating on any of the coated steel panels exposed to the salt spray for 1000 h. However, many corrosion spots were noticed on the steel panels coated with the zinc-rich epoxy steel primer. A few corrosion spots were noted on the steel panels coated with the cement-based epoxy coating. However, the coating remained intact after exposure to the salt spray. A rating of 10 and a rust grade of 2 (0% rust) were assigned to the specimens coated with the cement-based epoxy coating, and the values of 10 and 2 (33% rust) were assigned to the specimens coated with the zinc-rich epoxy coating.
ii. The time to crack of concrete specimens, due to the accelerated corrosion of steel bars coated with the cement-based coating, was longer than the specimens coated with the zinc-rich epoxy coating. The average time to crack in the concrete specimens with uncoated steel bars was 113 h, while it was 282 and 110 h, respectively, in the bars coated with cement-based and zinc-rich epoxy coatings.
iii. The corrosion potentials on steel in the slab specimens did not indicate the formation of incipient anodes at the junction of old and new concrete. The average corrosion potential in the uncoated specimens was more negative than −350 mV CSE (ASTM C876 threshold value) compared to the average potentials on steel coated with the cement-based epoxy resin or zinc-rich epoxy. The corrosion potentials on steel coated with cement-based epoxy resins and zinc-rich epoxy coatings were almost similar.
iv. Based on the results of the tested coatings in salt spray exposure and accelerated corrosion, it may be concluded that the cement-based coating performs better than the zinc-rich coating. There may be a decrease in the bond between steel and concrete. However, the decrease in the bond will be within the allowable value.

## Figures and Tables

**Table 1:** Details of the used coatings according to the manufacturer’s specifications.

Coating Type	Composition
Cement-based coating	Cementitious, three-component epoxy resin
Zinc-rich epoxy coating	Zinc primer, supplied as a single component grey-colored liquid based on metallic zinc and epoxy resin

**Table 2:** Description of the prepared laboratory and field specimens.

Specimen ID			Details
L-UnC-1	L-UnC-2	L-UnC-3	Lab cylindrical specimens with uncoated steel bars
F-UnC-1	F-UnC-2	F-UnC-3	Field slab specimens with uncoated steel bars
L-CmC-1	L-CmC-2	L-CmC-3	Lab cylindrical specimens with cement-based coating on the steel bars
F-CmC-1	F-CmC-2	F-CmC-3	Field slab specimen with cement-based coating on the steel bars
L-ZnC-1	L-ZnC-2	L-ZnC-3	Lab cylindrical specimens with zinc-rich epoxy coating on the steel bars
F-ZnC-1	F-ZnC-2	F-ZnC-3	Field slab specimens with zinc-rich epoxy coating on the steel bars

**Table 3:** Test conditions.

Details	Value
Salt solution	5% NaCl
pH of the salt solution	6.8 to 7.2
Air supply	20 psi
Chamber temperature	35 ± 1 °C
Period of exposure	1000 h
Relative humidity	80%

**Table 6:** Time to crack initiation in the concrete specimens with uncoated and coated steel bars.

Specimen Designation	Time to Crack, h	Average Time to Crack, h
L-UnC-1	135	113
L-UnC-2	130	
L-UnC-3	75	
L-CmC-1	240	282
L-CmC-2	265	
L-CmC-3	340	
L-ZnC-1	75	110
L-ZnC-2	125	
L-ZnC-3	130	

## Data Availability

Data available on request.
